# Assessing the Efficacy of ChatGPT in Solving Questions Based on the Core Concepts in Physiology

**DOI:** 10.7759/cureus.43314

**Published:** 2023-08-10

**Authors:** Arijita Banerjee, Aquil Ahmad, Payal Bhalla, Kavita Goyal

**Affiliations:** 1 Physiology, Indian Institute of Technology Kharagpur, Kharagpur, IND; 2 Physiology, Maulana Azad Medical College, New Delhi, IND; 3 Physiology, Shri Atal Bihari Vajpayee Medical College, Faridabad, IND

**Keywords:** artificial intelligence (ai), transfer of learning, core concepts, medical physiology, chat gpt

## Abstract

Background and objective

ChatGPT is a large language model (LLM) generative artificial intelligence (AI) chatbot trained through deep learning to produce human-like language skills and analysis of simple problems across a wide variety of subject areas. However, in terms of facilitating the transfer of learning in medical education, a concern has arisen that while AI is adept at applying surface-level understanding, it does not have the necessary in-depth knowledge to act at an expert level, particularly in addressing the core concepts. In this study, we explored the efficacy of ChatGPT in solving various reasoning questions based on the five core concepts applied to different modules in the subject of physiology.

Materials and methods

In this study, a total of 82 reasoning-type questions from six modules applicable to the five core concepts were created by the subject experts. The questions were used to chat with the conversational AI tool and the responses generated at first instance were considered for scoring and analysis. To compare the scores among various modules and five core concepts separately, the Kruskal-Wallis test along with post hoc analysis were used.

Results

The overall mean score for the modules (60 questions) was 3.72 ±0.26 while the average score obtained for the core concepts (60 questions) was 3.68 ±0.30. Furthermore, statistically significant differences (p=0.05 for modules and p=0.024 for core concepts) were observed among various modules as well as core concepts.

Conclusion

The significant differences observed in the scores among various modules and core concepts highlight the varying execution of the same software tool, thereby necessitating the need for further evaluation of AI-enabled learning applications to enhance the transfer of learning among undergraduates.

## Introduction

ChatGPT is a large language model (LLM) generative artificial intelligence (AI) chatbot trained via deep learning to produce human-like language and analysis of simple problems across a wide variety of subject areas. GPT stands for Generative Pretrained Transformer, which has been trained by machine learning to use its cognitive skills in addressing any given query and coming up with instant responses. The chatbot named ChatGPT was introduced in 2022 by OpenAI. It is a free-to-use application, which actually has increased its wider acceptability across the world. This AI application has the potential to transform the higher education system in terms of research, value-added learning, and publishing [1-3].

Medical students often fail to translate previously learned concepts to new applications; for example, students that learn the basic principles of hemodynamics in cardiovascular physiology struggle to apply the same concepts to gas exchange and airflow in the pulmonary system. As stated by Michael and Farland, focusing on the core concepts can augment the transfer of conceptual learning from one physiological system to the other. The intrinsic ability to understand and acknowledge that something which is learned earlier can later be applicable to something novel is a precious and powerful skill in the field of physiology where the learning is vast and ever-expanding. Five core concepts, namely, ﬂow down gradients, cell-to-cell communication, homeostasis, cell membrane, and mass balance have been developed and validated by Michael and several others. Competency-based medical education (CBME) in India is mainly focused on acquiring certain competencies that require a conceptual understanding of various topics rather than just memorizing them. As per the National Medical Commission (NMC), the physiology curriculum consists of a total of 11 modules to assess the competency of first-year medical graduates [4-6].

A majority of the studies using ChatGPT have applied this design to standardized, multiple-choice questions. In this study, we tried to explore the efficacy of ChatGPT in solving various reasoning questions based on the five core concepts applied to different modules in the subject of physiology. Further core concepts are highlighted to help students and faculty for developing effective educational tools in physiology for the transfer of learning.

## Materials and methods

Study design

This was a cross-sectional observational study conducted in the Department of Physiology, Dr. B.C Roy Multispeciality Medical Research Centre, IIT Kharagpur during the period of May-June 2023.

Study tool

OpenAI's free version of ChatGPT (version 3.5) was used for our study.

Ethical consideration

Ethical approval was not required for this study since it did not involve any human or animal research participants.

Core concepts: preparation of questions

As per the NMC Physiology syllabus, six modules were randomly selected by three professor-level subject experts in physiology with more than 12 years of experience in the field, and each of them prepared two reasoning-type questions pertaining to each of the five core concepts in all six modules. In this way, a total of 82 reasoning-type questions applicable to the five core concepts were created. The content validity of the questions was examined by the above-mentioned subject experts. Both of them examined the compatibility of each question in relation to knowledge, concepts, and application of the course content. The answer key to all the reasoning questions was prepared before the beginning of the test on ChatGPT. The various modules incorporated in this study and the applicability of five core concepts to all these modules are summarized in Table 1.

**Table 1:** Physiology core concepts in various modules

Modules/core concepts	Homeostasis	Flow down gradients	Cell membrane	Cell-to-cell communication	Mass balance
Cardiovascular physiology	Regulation of mean arterial pressure. Sympathovagal balance	Hemodynamics. Capillary exchange	Voltage-gated calcium channels. Adrenergic and cholinergic receptors	Gap junctions. Excitation-contraction coupling	Cardiac action potential. Pressure-volume loops
Neurophysiology	Regulation of posture. Sensory mechanisms/reflexes	Saltatory conduction. Axonal flow	Voltage-gated sodium and potassium channels. Receptors	Synaptic transmission. Skeletal muscle contraction	Resting membrane potential. Action potential
Respiratory physiology	Regulation of respiration. Acid-base balance	Exchange of gases. Closing volume	Respiratory membrane. Alveolar diffusion	Diffusion of gases. Ventilation/perfusion ratio	Alveolar gas equations. High altitude and space physiology
Gastrointestinal physiology	Glucose absorption and regulation. Iron regulation	Absorption in the small intestine. Movement of chyme	Smooth muscle. Acid peptic disease	Gastric acid secretion. Gastrointestinal hormones secretion	Substrate in pancreatic juice. Slow wave potentials
Renal physiology	Renin-angiotensin-aldosterone mechanism. Regulation of sodium and potassium ions in extracellular fluid	Glomerular fitration. Concentration of urine	Transporters. Aquaporins	Role of antidiuretic hormone on collecting duct. Sodium absorption in renal tubules	Total body water distribution. Renal clearance
Endocrine physiology	Glucose regulation. Calcium regulation	Hormone transport. Hormone secretion	Second messengers. Receptors	Signal transduction. Hypothalamo-pituitary-adrenal axis	Free thyroid hormone concentration. Free testosterone levels

Data collection

The questions were used to chat with the conversational AI tool and the responses generated at first instance were considered for scoring and analysis.

Data analysis

All the questions were checked separately by the three subject experts based on the prepared answer key, which meant that each question had three scores. The scoring was implemented based on a rating scale of 0-5, where 0 meant incorrect/irrelevant response and 5 signified an absolutely correct answer. The average of the three scores was taken into account. Data was incorporated using IBM SPSS Statistics software version 22.0 (IBM Corp., Armonk, NY). Descriptive statistical analysis was done and data was expressed in terms of mean and standard deviation (SD). Since the data was not distributed normally, the one-sample median test was implemented to check for the precision of the response using a hypothetical expected value of 5. To compare the scores among various modules and five core concepts separately, the Kruskal-Wallis test along with post hoc analysis were used. P-values less than or equal to 0.05 were considered statistically significant.

## Results

In our study, we analyzed the scores of modules and core concepts separately. The overall mean score for the modules (60 questions) was 3.72 ±0.26 while the average score for the core concepts (60 questions) was 3.68 ±0.30. The average scores for all the modules are presented in Table 2: the highest score was obtained in cardiovascular physiology (3.82 ±0.33), followed by neurophysiology (3.74 ±0.21), and the lowest score was obtained in endocrine physiology (3.62 ±0.16). The median values of the scores were significantly different from our hypothetical values in almost all the modules (p<0.001). The average median score for all the modules was 3.86 ±0.24, giving an accuracy of 77%.

**Table 2:** Module-wise average scores for the total 6 modules N: number of questions; P: physiology; SD: standard deviation; Q1-Q3: interquartile range

One-sample median test	Cardio-P (N=10)	Neuro-P (N=10)		Respiratory-P (N=10)	Gastro-P (N=10)		Renal-P (N=10)	Endocrine-P (N=10)	
Hypothetical median	5	5		5	5		5	5	
Mean	3.82	3.74		3.67	3.70		3.72	3.62	
SD	0.33	0.21		0.21	0.26		0.26	0.16	
Median (Q1-Q3)	4.00 (3.50-4.5)	3.75 (3.16-4.16)		3.83 (3.00-3.83)	3.83 (3.5-4.00)		3.92 (3.50-4.16)	3.83 (3.16-4.00)	
P-value	0.03		<0.001	<0.001		<0.001	0.002		<0.001

**Figure 1:**
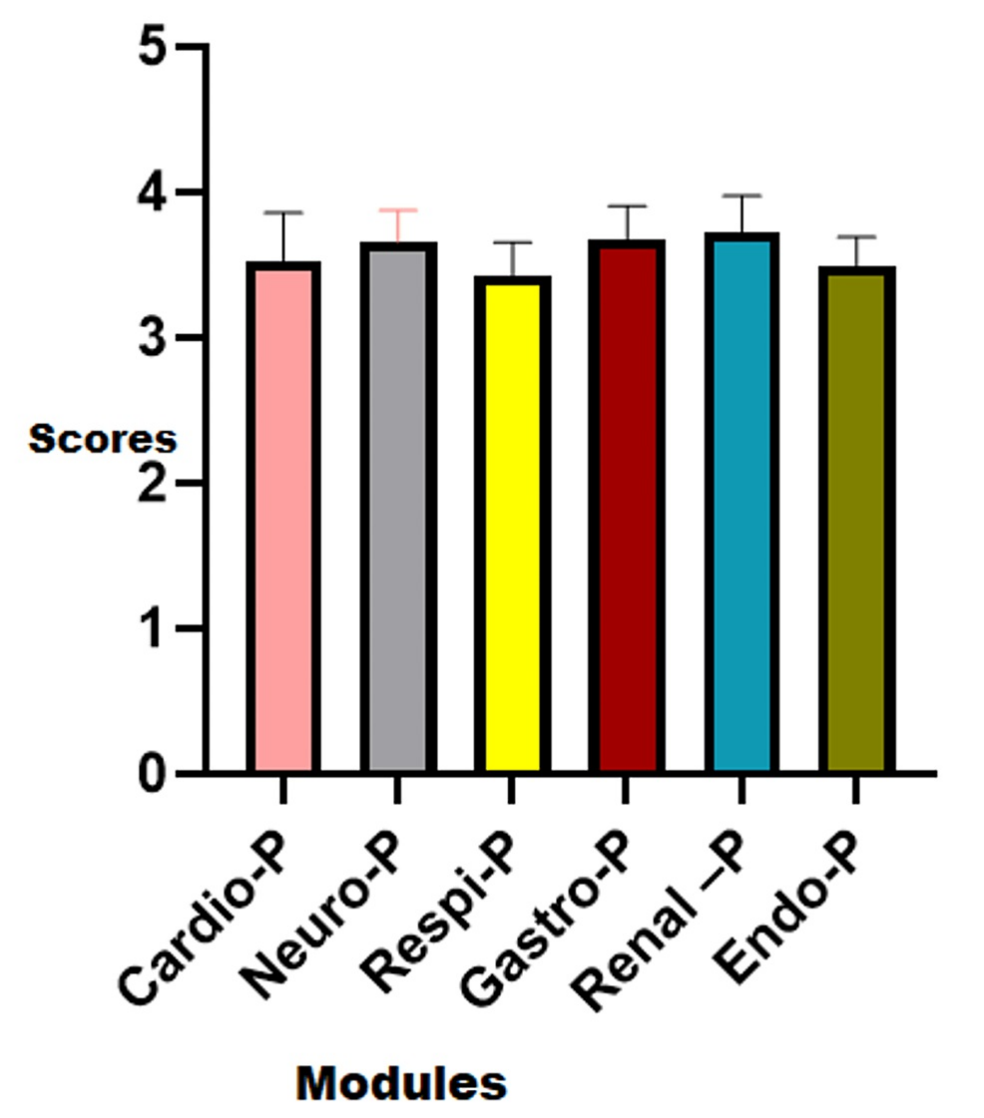
Comparison of scoring among various modules P: physiology

**Figure 2:**
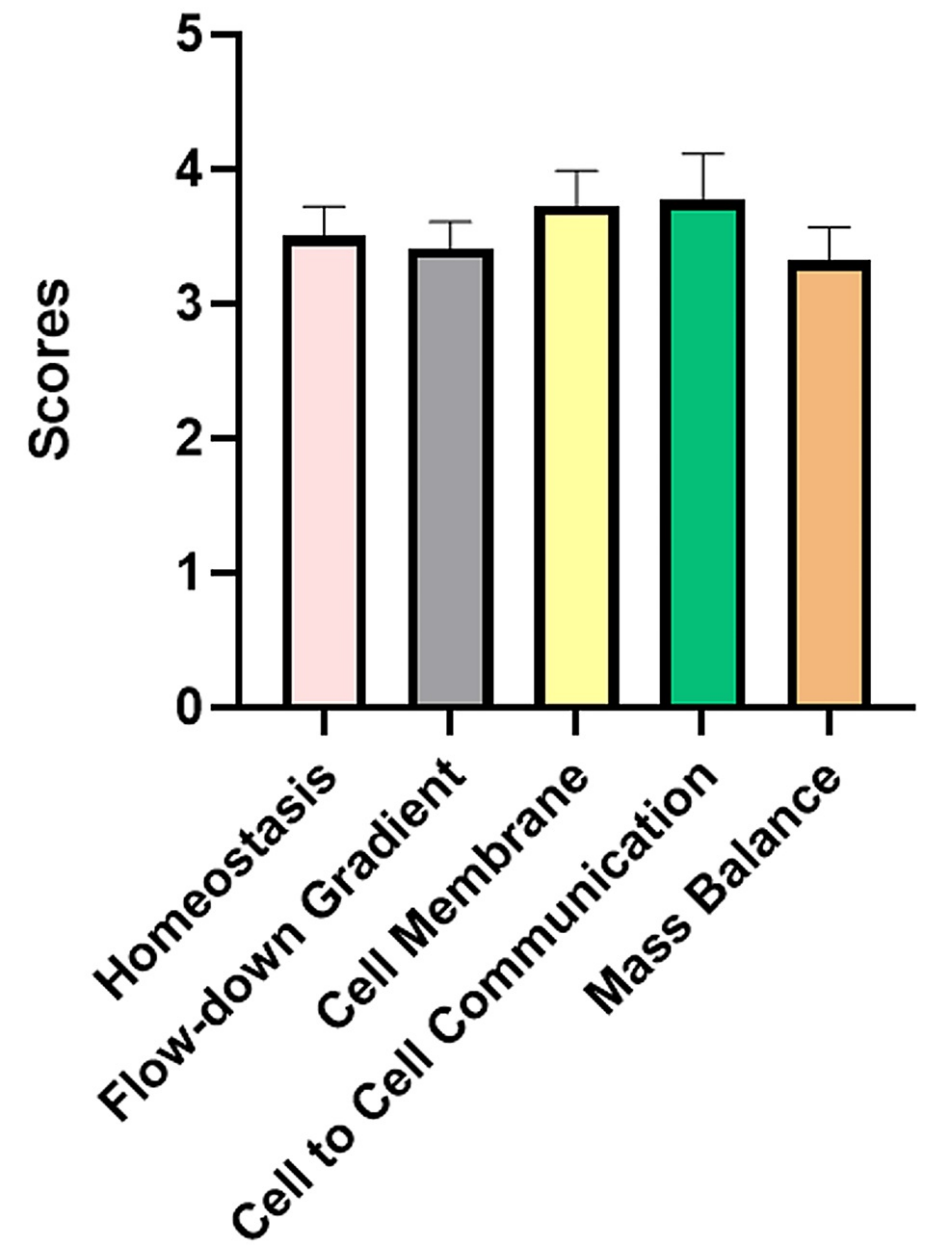
Comparison of scoring among core concepts

After performing Kruskal-Wallis analysis for both module-wise scoring and core concepts-wise scoring, a statistically significant difference (p=0.05 for modules and p=0.024 for core concepts) was found among various modules as well as core concepts, which means that the conversational AI software application was different among the modules as well as the core concepts, as depicted in Figures 1, 2. The significant differences observed in the scores among various modules and core concepts indicate the varying execution of the same software tool and thus highlight the need for further evaluation of AI-enabled learning applications.

## Discussion

When information or skills learned are applied to a new context, it is known as transfer of learning. It is an integral part of the process of learning. As stated by Perkins and Salomon, the transfer of learning occurs when learning from one context has an impact on another context or situation. A few authors have mentioned the concept of frame transfer along two different dimensions, with regard to teaching about the transfer of learning [7].

**Figure 3:**
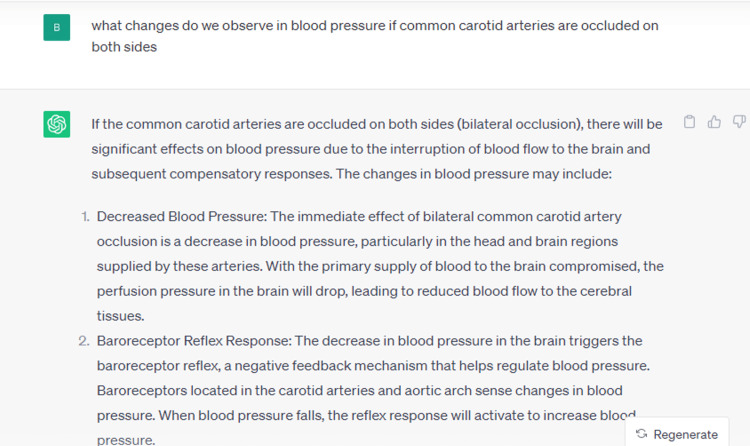
An example of the answer provided by ChatGPT (cardiovascular physiology-homeostasis)

In our present study, it was observed that the accuracy of ChatGPT in interpreting physiology core concepts was 77%. To date, there is no evidence of any other studies related to ChatGPT and physiology core concepts; however, a few studies related to microbiology and pathology have analyzed the use of ChatGPT in those fields, and they have found an accuracy rate of approximately 80%. Based on our findings, the overall mean score for the modules (60 questions) and core concepts (60 questions) were 3.72 ±0.26 and 3.68 ±0.30 respectively, indicating a score of less than 4.00, which warrants further training of the machine learning-based ChatGPT. Out of six modules, scores were comparatively higher in cardiovascular physiology followed by neurophysiology. The differences in the scores among various modules might be due to the restraint in tutoring the AI application. Among the core concepts, the highest score was achieved in "cell-to-cell communications" followed by "cell membrane" while the lowest score was obtained in "mass balance". The significant differences observed in the scores among various modules and core concepts highlight the varying execution of the same software tool, and excessively elaborative answers, and hence emphasize the need for further evaluation of AI-enabled learning applications. An example of the response given by ChatGPT is shown in Figure 3. Let us consider the example of "homeostasis". Undergraduate students can learn from the textbooks that Walter Cannon coined the term, and various types of mechanisms bring about homeostasis, like negative feedback, positive feedback, and feedforward mechanisms. Yet, they are not taught the basic model of homeostasis, which consists of "control center", "controller", "effector", "error detector", "error signal", "controlled variable", "sensed variable", "set point", or "gain" [8-12].

Transfer of learning is a difﬁcult and complex process. It is also important to note that the transfer does not occur naturally but requires continuous effort. If the initial learning is in-depth and well understood, and generalized, the transfer becomes easy. Similarities between the initial learning and new context also aid in the transfer as compared to unfamiliar topics or contexts. This becomes more of a challenge in physiology courses, as the textbooks or the routine classroom practices make the transfer a less likely phenomenon. Hence, our present study has depicted further modifications and training methods for the ChatGPT tool, which are required to enhance this transfer of learning among undergraduate students [12-15].

This study has a few limitations. Our analysis was limited to one subject, physiology, and hence the findings may not be generalizable to other subjects; also, some evaluation bias may have crept in due to the subjective scribing by the three faculty members. Also, in our study, the ChatGPT 3.5 version (free version) was used to assess reasoning skills instead of the ChatGPT 4.0 version (paid version).

## Conclusions

In order to facilitate the transfer of learning in physiology, it is important to realize that the students will transfer what they have learned. Core concepts form the building blocks of such knowledge, which needs to be more precise and accurate while using AI-enabled learning applications. Based on our findings, the ChatGPT tool needs more training with further data since the performance of the same is limited by input. Further research is needed to explore the current paid version of ChatGPT (version 4.0).

## References

[1] Kung TH, Cheatham M, Medenilla A (2023). Performance of ChatGPT on USMLE: potential for AI-assisted medical education using large language models. PLOS Digit Health.

[2] Huh S (2023). Are ChatGPT’s knowledge and interpretation ability comparable to those of medical students in Korea for taking a parasitology examination? A descriptive study. J Educ Eval Health Prof.

[3] Shah N, Desai C, Jorwekar G, Badyal D, Singh T (2016). Competency-based medical education: an overview and application in pharmacology. Indian J Pharmacol.

[4] Sinha RK, Deb Roy A, Kumar N, Mondal H (2023). Applicability of Chat-GPT in assisting to solve higher order problems in pathology. Cureus.

[5] Okonkwo CW, Ade-Ibijola A (2021). Chatbots applications in education: a systematic review. Computers Educ Artif Intell.

[6] Arif TB, Munaf U, Ul-Haque I (2023). The future of medical education and research: is ChatGPT a blessing or blight in disguise?. Med Educ Online.

[7] Hull K, Jensen M, Gerrits R, Ross KT (2017). Core concepts for anatomy and physiology: a paradigm shift in course and curriculum design. HAPS Educ.

[8] Michael J, McFarland J (2011). The core principles ("big ideas") of physiology: results of faculty surveys. Adv Physiol Educ.

[9] Michael J, Modell H (2019). A conceptual framework for the core concept of "cell membrane". Adv Physiol Educ.

[10] McFarland JL, Michael JA (2020). Reflections on core concepts for undergraduate physiology programs. Adv Physiol Educ.

[11] Michael J (2007). Conceptual assessment in the biological sciences: a National Science Foundation-sponsored workshop. Adv Physiol Educ.

[12] Modell HI (2000). How to help students understand physiology? Emphasize general models. Adv Physiol Educ.

[13] Michael J (2007). What makes physiology hard for students to learn? Results of a faculty survey. Adv Physiol Educ.

[14] Minasian-Batmanian L (2003). An innovative, interactive, self-instructional, online alternative to replace a face-to-face respiratory control practical. Br J Educ Technol.

[15] Minasian-Batmanian LC (2002). Guidelines for developing an online learning strategy for your subject. Med Teach.

